# The effectiveness of Tai Chi on the physical and psychological well-being of college students: a study protocol for a randomized controlled trial

**DOI:** 10.1186/1745-6215-15-129

**Published:** 2014-04-17

**Authors:** Guohua Zheng, Xiulu Lan, Moyi Li, Kun Ling, Hui Lin, Lidian Chen, Jing Tao, Junzhe Li, Xin Zheng, Bai Chen, Qianying Fang

**Affiliations:** 1 Academy of Integrative Medicine, Fujian University of Traditional Chinese Medicine, HuaTuo Road, Fuzhou 350122, China; 2 Rehabilitation Medicine College, Fujian University of Traditional Chinese Medicine, HuaTuo Road, Fuzhou 350122, China; 3 Department of Physical Education, Fujian University of Traditional Chinese Medicine, HuaTuo Road, Fuzhou 350122, China; 4 Fujian University of Traditional Chinese Medicine, HuaTuo Road, Fuzhou 350122, China

**Keywords:** Tai Chi Chuan, College students, Psychological well-being, Physical health

## Abstract

**Background:**

The physical and mental health of college-age youths tends to continuously decline around the world. It is therefore important to promote health during this period. As a traditional Chinese mind-body exercise, Tai Chi Chuan (TCC) may be an available selection. However for the college student population, the evidence is unclear as to whether TCC can be recommended as an effective exercise for promoting their physical and psychological wellbeing. Therefore high quality, rigorous, prospective, and well-controlled randomized trials are needed to further understand TCC serving as a psychological and physical intervention in college age populations.

**Method/Design:**

We designed a randomized, single-blind, parallel-controlled trial with a sample size of 206 participants. All the participants who meet the inclusion criteria come from Fujian University of Traditional Chinese Medicine (FJTCM). Participants of the TCC training group will receive TCC training at a frequency of five days per week for one hour per day for 12 weeks. No specific exercise will be administered on the participants in the control group. Both physical and mental health outcomes, including balance ability, lower limb proprioception, flexibility, physical fitness, self-efficacy, psychological symptoms, attention span, stress, self-esteem, mood and mindfulness, quality of life, and quality of sleep. Safety outcomes will be evaluated by blinded operators at baseline, 12 and 24-weeks post-intervention.

**Discussion:**

This protocol presents an objective design of a randomized, single-blind trial that will test the effectiveness and safety of TCC on the physical and psychological wellbeing of college students. If the outcome is positive, the results will provide higher quality evidence of TCC on the physical and mental health of college age populations.

**Trial registration:**

Chinese Clinical Trial Registry: ChiCTR-TRC-13003328.

## Background

College students represent the future of families, communities, and countries. The college age is also a critical time in which individuals begin to take definitive steps towards independence, and is considered to be the first major transition an individual faces [1]. However, due to the intense pressure of competition among students, their physical activity decline is evident during the first transition period into adulthood, especially in the first college year [2-4]. It is estimated that only 44% of college age students in America meet physical activity recommendations, as opposed to 68% of high school students [5]. Similarly, the percentage of physically inactive college students is 13.5% for Taiwan, 16.8% for Hong Kong, and 28.5% for Korea [6]. It is well know that physical activity can enhance the functioning of cardiovascular systems and have substantial benefits in reducing the risk of cardiovascular diseases [7,8]. College students may therefore be a population at risk and susceptible to chronic diseases [9-11].

The psychological and/or mental wellbeing of college students might be ‘worse off’ than that of the general population. As a result of risky behaviors and multiple stressors, such as academic challenge, competition and achievements [12], various forms of mental health problems are more frequently present among college students than same-aged non student populations [13-15]. Convincing evidence has shown a marked increase in psychological distress including depression, paranoia, and hypomania among American college students over the past 50 years [16]. Longitudinal studies of psychological distress in college students showed that, although distress levels peaked during the first year and then declined for most students, some of them manifested with severe distress levels which did not decrease over time [17,18]. If left ignored and untreated, mental health problems may lead to students dropping out of college, attempting or committing suicide, or engaging in other risky, dangerous behaviors [19]. However, it is estimated that only a minority of college students with mental health problems seek and receive adequate help [20].

The growing research continues to strengthen the idea that regular exercise or physical activities are positively associated with physical and psychological health outcomes [21,22]. As a traditional Chinese mind-body exercise, TCC has been practiced for many centuries in China and is increasing in popularity in the West. Through deeply diaphragmatic breathing, basic slow and gentle mind-body moments, practitioners can achieve an efficiency of ‘body relaxation and mind calm’ and Tian Ren He Yi (the theory that mankind is an integral part of nature) [23]. TCC, or meditative movement types of exercise in general may provide attractive and effective exercise alternatives for the large amount of people at a risk of preventable diseases, who live a sedentary lifestyle, and who lack the motivation to engage in more conventional exercise [24]. Several systematic reviews have suggested that TCC may have significant improvement in balance capability, flexibility, cardiovascular and respiratory function, hypertension, osteoporosis, depression, anxiety and stress of the general population [25-33]. However, it was difficult to draw firm conclusions due to the fact that limitations or biases exist in the majority of the studies, such as small sample size or lacking rigorously randomized design [34]. Furthermore, for young adults, (particularly the college student population) the evidence is unclear as to whether TCC can be recommended as an effective exercise for improving emotional state, psychological wellbeing and physical fitness. Therefore high quality, rigorous, prospective, well-controlled randomized trials with appropriate comparison groups and validated outcome measures are needed to further understand the effects of TCC serving as an intervention for specific psychological outcomes in college student populations. The purpose of this trial is to systematically evaluate the effects of TCC on physical and psychological outcomes of college students including mood, anxiety, psychological wellbeing, self-efficacy, and quality of life.

### Objective

We designed a strict randomized controlled trial to systematically evaluate the effectiveness of TCC exercise on the physical and mental health of college students.

## Methods/design

### Study design

**Figure 1:**
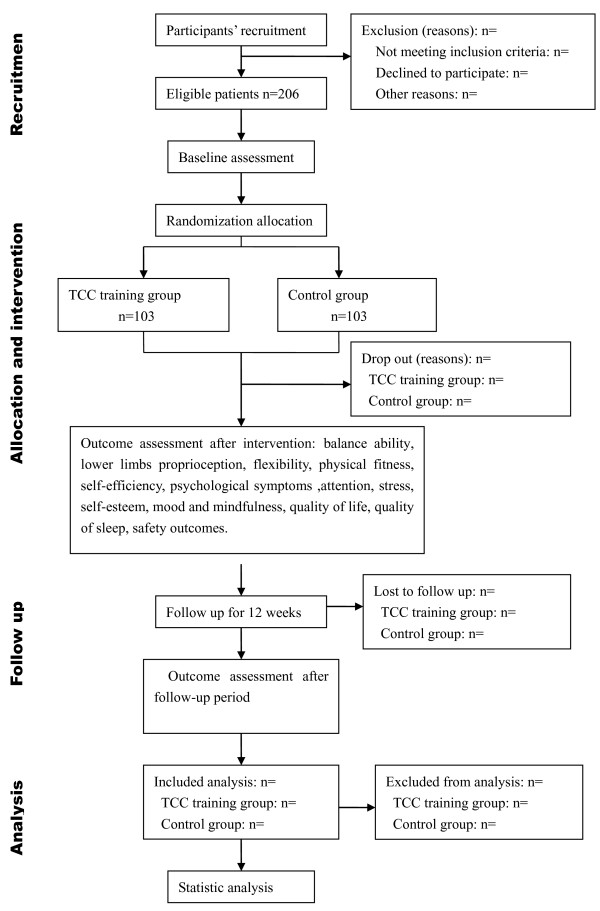
**Flow diagram of participants.** TCC, Tai Chi Chuan.

This study was designed as a randomized, parallel-controlled, single-blinded (assessor and statistician) trial. The allocation of participants will be equal (1:1) to the TCC exercise group (intervention group) and the control group. All assessment will be conducted by the blinded assessors at Fujian University of Traditional Chinese Medicine Affiliated Rehabilitation Hospital, and the gymnasium of FJTCM. The TCC exercise will be conducted at the gymnasium of Fujian University of TCM (Traditional Chinese Medicine), and instructed by two qualified coaches. The entire trial program is illustrated in Figure 1.

### Sample size estimation

Sample size estimation in this RCT (Randomized Controlled Trial) is based on the expected improvement of balance ability in 12-weeks of TCC exercise in college students. The data of our preliminary experiment showed that the means and standard deviation of the balance ability (presented by motion of ellipse area which was tested by standing with eyes closed on the flat of the Proprioceptive Kinetics system) in the participants was 531.25 and 173.78. 15% respectively. Improvement of balance ability will be expected after the 12 week-intervention. Improvement will be measured according to the same size of the estimation formula:

(1) $$\begin{array}{ll} n_{1} & =n_{2}\\ & =2{\left(\frac{z_{\alpha /2}+z_{\beta }}{M_{e}-M_{c}}\right)}^{2}\delta^{2}\;\left(\text{Set}\;\alpha =0.05,\beta =0.10\right). \end{array}$$

It is estimated that a sample size of 93 participants per group will be required, considering a 10% dropout and exit rate. Therefore, we will recruit an approximate total of 206 participants, with 103 participants in each group.

### Participants and recruitment

A total of 206 participants will be recruited at FJTCM. We plan to advertise the recruitment program through advertisements on the campus bulletin board and via campus radio. Interested students will contact the research assistants and will be screened according to the inclusion and exclusion criteria. The potential participants will be required to sign an informed consent agreement if they fulfill inclusion criteria and do not have any exclusion criteria, before enrollment in this trial.

### Inclusion criteria

Participants have to fulfill the following criteria: aged between 16 and 25 years, a full time freshman or sophomore, and have provided written informed consent for participation in the trial.

### Exclusion criteria

Students would be ineligible if they meet any of the following conditions: are engaged in long term exercising TCC or other Tai Chi derived movements, are a member of the Students’ Wushu Association, Taekwondo Association, Aerobic Association or Dance Association, or suffer from severe cardiovascular or musculoskeletal disease.

### Withdrawal criteria

Participants will be withdrawn from the trial if they present any of the following conditions: poor compliance (mean compliance < 85% at the last estimation) or noncompliance, occurrence of a serious adverse event, initiative exit are unable to progress because of sudden disease, members in the control group have regularly engaged in TCC exercise.

### Ethical consideration

This study protocol adheres to the principles of the Declaration of Helsinki and has been approved by the ethics committee of FJTCM (No. 042). Written consent will be obtained from each participant before the baseline assessment. All participants will have the right to withdraw from the study at any time.

### Randomization and allocation concealment

Participants will be randomly allocated to the TCC training group or the control group. Randomization will be performed at post-baseline assessment. The randomization list will be generated via the Statistical Analysis System (SAS, version 9.1) by an independent non-investigator working in the Center for EBM(Evidence-Based Medicine) of Academy of Integrative Medicine Fujian China. The allocation sequence will be concealed using password restricted files which will remain in the care of the project manager. The eligible participants will be informed of the allocation result by the project manager after post-baseline assessment.

### Blinding

In this trial it is impossible to blind the participants and TCC coaches. Nevertheless, we will assign a specified project manager to be in charge of the random-allocated sequence and blind code of allocation in which the TCC exercise group or control group will be replaced by the letter A or B. In addition, we will define the obligations of each investigator; the project manager and TCC coaches will not take part in the assessment outcome; and the outcome assessors and the statistical analyzer will not be involved in the participants’ recruitment and allocating. The allocation sequence and blind codes will be preserved by an independent project manager until the statistical analysis is completed.

### Intervention

#### The TCC training group

**Figure 2:**
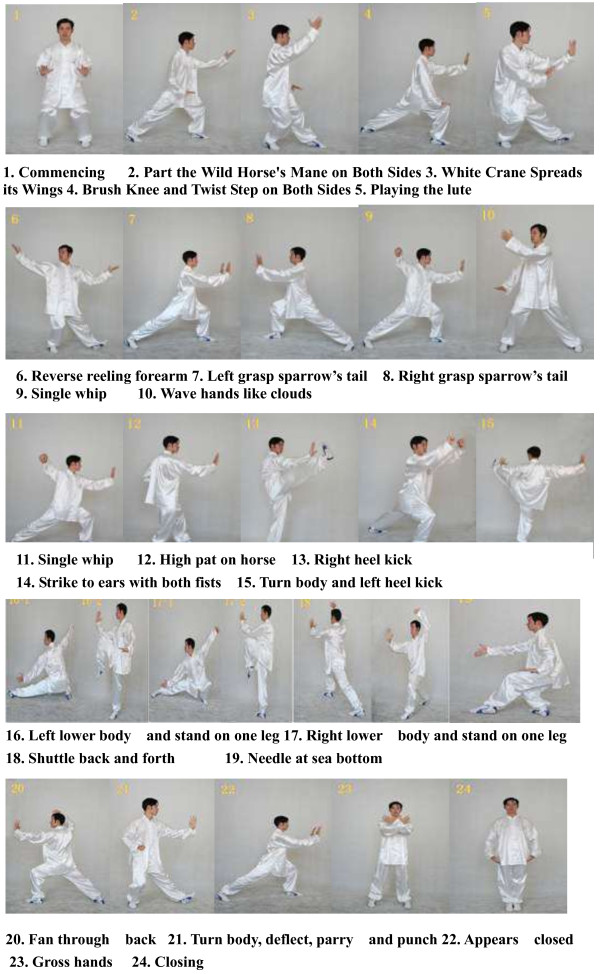
24 forms simplified Tai Chi Chuan.

Participants allocated to the TCC training groups will receive 12 weeks of TCC training. The TCC training will be instructed by two experienced TCC coaches who have been qualified and engaged in the teaching of TCC course for at least 15 years. The 24 forms of simplified TCC, which are recommended as a popular health sport by the General Administration of Sport of China [35,36], will be applied to members of the intervention group. Participants of the TCC training group will be gathered at the campus gymnasium to exercise together at a frequency of five days per week. TCC training will be performed for 60 minutes per day, and will also include 10 minutes of warm-up (for example weight shifting, arm swinging, gentle stretches of the neck). Participants will be taught the ‘meditation through movement’ art of TCC, which includes the rationale that TCC constitutes a health benefit, the action essence of TCC, the TCM philosophy opinion of TCC, and the deep breathing method. The instruction of TCC will be both verbal and visual. The systematic approach has been designed by two coaches and can be viewed in Figure 2.

#### Control group

No specific exercise will be administered on the participants in the control group. They will be informed to maintain their original lifestyle.

The intervention period in this trial will last 12 weeks. All participants will be required to record physical activity diaries, including the type and intensity of physical activity or exercises, as well as the sedentary time and sleeping time everyday throughout this study.

### Follow up period

During the 12-week follow up period, all of the participants will return to their original lifestyles, but be required to record their daily physical activities or sport information. The primary outcomes will be re-measured at end of follow up period.

### Outcome measurement

The primary and secondary outcomes will be tested at baseline, 12 and 24-week post-intervention. A schedule of assessments that will be carried out is listed in Table 1. The balance ability and lower limbs proprioception will be assessed at the Rehabilitation Hospital Affiliated to FJTCM by three experienced rehabilitation therapists who are not otherwise involved in this study. The physical fitness test, cardiopulmonary function, flexibility, vital capacity, and weight will be conducted at the campus gymnasium by the physical education teachers. The outcome assessors of this trial will be in charge of the assessment of relevant psychology scales including self-efficacy, attention span, stress, self-esteem, and quality of sleep.

**Table 1:** Trial processes chart

**Items**	**Before enrollment (week)**	**TCC training phase (week)**	**Training end (week)**	**Follow up (week)**	**Follow up end (week)**
	**-2-(-1)**	**1-12**	**13**	**13-24**	**25**
Inclusion criteria	*				
Exclusion criteria	*				
Informed consent	*				
Baseline	*				
Randomization	*				
Self-esteem	*		*		*
Mood and mindfulness	*		*		*
Self-efficacy	*		*		*
Psychological symptom score	*		*		*
Stress test	*		*		*
Flexibility	*		*		*
Grip strength	*		*		*
Step testing	*		*		*
Rest heart rate	*		*		*
Vital capacity	*		*		*
Systolic blood pressure	*		*		*
Diastolic blood pressure	*		*		*
Balance	*		*		*
Lower limbs proprioception	*		*		*
Attention span	*		*		*
Quality of life	*		*		*
Quality of sleep	*		*		*
Safety outcomes		*		*	
Self-report diaries		*		*	

TCC, Tai Chi Chuan.

*The process should be conducted in this trial.

### Primary outcome measures

Balance ability will be tested by standing with eyes open for 30 seconds and standing with eyes closed for 30 seconds via the Pro-kin system (product type: PK254P) produced by Tecnobody .S.r.l company, Italy [37,38]. Lower limbs proprioception in both left and right legs will be tested via the Pro-kin system [39]. Flexibility will be tested with Sit and Reach flexibility test equipment (Machine type: CSTF-TQ-5000, Zhongtitongfang Co., Ltd., Beijing, China) Self-efficacy with be tested using the self-regulatory self-efficacy scale [40]. the psychological symptom score will be assessed by using the SCL-90 scale [41]. Attention span will be assessed using the Schulte grid (8*8) scale. Stress will be measured using the Chinese Perceived Stress Scale [42].

### Secondary outcomes measures

Cardiopulmonary function will be evaluated indirectly by a step test, vital capacity, blood pressure and rest heart rate. Step testing will be conducted by an electronic step test instrument (stairs with a 30 cm step height will be used for males, and 25 cm for females) (Machine type: Beijing ZhongTi Tongfang Co., Ltd., model CSTF-TZ-5000). Vital Capacity will be tested by an electronic vital capacity instrument (Machine type: Beijing ZhongTi Tongfang Co., Ltd., model CSTF-FH-5000). Blood pressure and rest heart rate will be tested by electric sphygmomanometers produced by the Omron Corporation, Dalian, China (product type: HEM-746C). Self-esteem will be assessed with self-esteem scale [43]. Mood and mindfulness will be measured with the Profile of Mood States (POMS) [44]. Quality of life will be tested using the WHOQOL-BREF scale [45], and finally, sleep quality will be measured using the Pittsburgh Sleep Quality Index (PSQI).

### Safety evaluation

Any adverse events (defined as any functional lesion caused by the intervention, such as knee joint or ankle sprain, knee soreness, lumbar muscle strain and so on) will be recorded on a case report form (CRF) during the intervention period. If any adverse event occurs, the coaches or project managers will provide the corresponding treatment to the participant. The adverse events will be immediately reported to the primary investigator and ethics committee to decide if the participant needs to withdraw from the trial.

### Statistical analyses plan

The primary outcomes of participants who are randomized and receive at least one treatment week will be carried out using the intention-to-treat (ITT) analysis. Per-protocol subject analysis (PPS) of the primary outcomes will include participants who have completed the 24-week study without major protocol violations and have a compliance rate of > 85%. We will compare the results of the ITT with that PP analysis to check whether the results are consistent or not. The continuous variables will be expressed using means with standard deviations or medians with ranges. For the variables with a normal distribution, statistical comparisons between the groups will be made by using a *t* test. If the variables have a non-normal distribution of ordinal level, statistical comparison between groups will be made using the Mann–Whitney *U* test. Measures with a discrete distribution will be expressed as percentages and analyzed by the *χ*^2^ or Fisher’s exact test as appropriate. A general linear model or logistic regression model will be applied to adjust the confounding influence if necessary.

All statistical tests will be performed using IBM SPSS 21.0 (version 21.0, IBM Corp., New York, NY, United States) with bilateral inspection. A *p* value of less than or equal to 0.05 will be considered as statistically insignificant.

## Discussion

The aim of this trial is to evaluate the effect of TCC on the physical and mental health of college students. The trial is supported by the ‘Traditional Chinese Exercises Program’ from the State Administration of Traditional Chinese Medicine.

The benefits of TCC on physical and psychological health have been reported in numerous studies, however, most of them pay attention to various patients or the aged population [24,25,29,30]. Furthermore, due to the poor quality of current studies, there is a lack of reliable evidence for TCC. In addition, currently few studies have reported on the association between TCC and the physical or mental health of college students.

We scrupulously designed this randomized, parallel-controlled, assessor-blinded and statistician-blinded trial to evaluate the effectiveness and safety of TCC on the physical and mental health of college students. Through a 12-week investigation with either TCC or control, results from a range of physical or psychological health assessments will provide clear information about differences in balance ability, flexibility, physical fitness, attention span self-efficacy, psychological symptoms, stress, mood and mindfulness, quality of life, quality of sleep and self-esteem between different groups. Changes of various variables before and after the intervention in this trial may strengthen evidence of TCC for the physical and psychological health of college students.

### Strengths and limitations

Initially, one of strengths of this study is that strict, complete, randomization and adequate concealment will be used in our trial. An independent non-investigator will conduct the randomization. Moreover, project manager will keep the allocation from the screeners, as well as outcome assessors and data analysts until the whole study term is done. Furthermore, we will rigorously perform quantity control to ensure the power. We will protect our participants’ information and outcomes by using password restricted files. Every training course will be supervised by a project manager to make sure coaches and participants perform every action seriously. We will also pay a return visit during the study to ensure participants record information in the diaries in timely and conscientiousness manner.

Secondly, the main limitation of this protocol is obvious. Ideally everyone involved in an RCT should be blinded but this is not always feasible in non-pharmacological trials [46]. Although it is impossible to carry out blinding to both the coaches and participants in this trial, we attempt to decrease possible bias by blinding both outcome assessors and data analysts.

In summary, we have developed a protocol with a randomized, parallel-controlled design to systematically evaluate the effectiveness of TCC for the physical and mental health of the college student population. If our study demonstrates a significant intervention effect, this would provide strong evidence for the application of TCC among the college student population.

## Trial status

Status at time of submission of this article: recruiting.

## Competing interests

The authors declare that they have no competing interests.

## Authors’ contributions

LDC, GHZ conceived of the idea, designed the study protocol, and XLL drafted the manuscript, GHZ revised it and write several sections of this manuscript. JT and GHZ are in charge of coordination and direct implementation. MYL will be the project manager assist to make sure the overall program successfully carries on, moreover KL, HL, JZL, XZ, BC, QYF are research assistants who will help organize to actively recruit, train, assess outcome, data manage and so on. All authors contributed to writing the manuscript and have read and approve the final manuscript for publication.
